# Immune Modulation by Human Secreted RNases at the Extracellular Space

**DOI:** 10.3389/fimmu.2018.01012

**Published:** 2018-05-16

**Authors:** Lu Lu, Jiarui Li, Mohammed Moussaoui, Ester Boix

**Affiliations:** Department of Biochemistry and Molecular Biology, Faculty of Biosciences, Universitat Autònoma de Barcelona, Cerdanyola del Vallès, Spain

**Keywords:** ribonucleases, innate immunity, RNA, extracellular, inflammation, infection

## Abstract

The ribonuclease A superfamily is a vertebrate-specific family of proteins that encompasses eight functional members in humans. The proteins are secreted by diverse innate immune cells, from blood cells to epithelial cells and their levels in our body fluids correlate with infection and inflammation processes. Recent studies ascribe a prominent role to secretory RNases in the extracellular space. Extracellular RNases endowed with immuno-modulatory and antimicrobial properties can participate in a wide variety of host defense tasks, from performing cellular housekeeping to maintaining body fluid sterility. Their expression and secretion are induced in response to a variety of injury stimuli. The secreted proteins can target damaged cells and facilitate their removal from the focus of infection or inflammation. Following tissue damage, RNases can participate in clearing RNA from cellular debris or work as signaling molecules to regulate the host response and contribute to tissue remodeling and repair. We provide here an overall perspective on the current knowledge of human RNases’ biological properties and their role in health and disease. The review also includes a brief description of other vertebrate family members and unrelated extracellular RNases that share common mechanisms of action. A better knowledge of RNase mechanism of actions and an understanding of their physiological roles should facilitate the development of novel therapeutics.

## Introduction

Thirty years ago Steven Benner conjectured the existence of extracellular RNA communicators (1, 2). At that time, he was investigating bovine RNaseA activity and expressed skepticism that pancreatic RNases merely removed the large amount of bacterial RNA present in the ruminant digestive tract (3, 4). Based on the diverse biological properties displayed by some RNaseA family members, i.e., anti-tumoural action, angiogenesis, and neurotoxicity, he suggested that the catalytic activity of vertebrate secreted RNases intervened in the regulation of the development of higher organisms. The hypothesis was launched well before the discovery of extracellular vesicles as horizontal nanovehicle carriers and well before the discovery that angiogenin, a member of the vertebrate-specific RNaseA superfamily, generates RNA regulatory fragments (5, 6). RNaseA, the vertebrate secretory RNases’ reference family member, is a small and highly stable protein that served as a working model for biochemists during the twentieth century; several Nobel prizes in chemistry were awarded for work with RNaseA (7, 8). Many times, researchers have tried unsuccessfully to rescue the RNaseA superfamily from its purely academic role (1, 7). Despite extensive knowledge on the mechanism of catalysis (8) and phylogeny (9–12), the biological properties of some family members remained puzzling, and the ultimate physiological roles of these proteins remained elusive. Exhaustive sequencing of RNases within vertebrates and a comparative phylogenetic analysis suggested that the family emerged with a host defense role (11, 13–16). Another interesting hypothesis suggested that granulocyte-secreted proteins could play a primary role in local tissue repair and the removal of macromolecular debris following cell damage during inflammation (17). The authors considered the potential contribution to RNA clearance by eosinophil secreted RNases. Later studies also indicated an RNA scavenging role for pancreatic-type RNases (18, 19). In addition, the secreted proteins displayed immuno-regulatory properties that suggest they could participate in the transmission and amplification of local danger signals (17, 20). Therefore, extracellular RNases are key players that ensure tissue health and body homeostasis. Indeed, we find examples of genetic deficiencies in extracellular RNases that lead to immune-related diseases, such as amyotrophic lateral sclerosis (ALS), associated with human RNase5 mutations (21, 22) and cystic leukoencephalopathy, a neuronal disorder associated with RNaseT2 deficiency (23, 24). Potential RNA-targeted therapeutic applications for secretory RNases were envisaged as far back as two decades ago (25–27).

Recent methodological advances in the cellular biology and RNA fields have facilitated novel approaches to understanding the *in vivo* role of extracellular RNases. Hopefully, knowledge on RNases action and trafficking in biological fluids will give path to translational research from academia to pharmaceutical industry. Indeed, recent experimental trials with animal models, such as those on hematopoiesis regulation by human RNase5 (28) or attenuation of extracellular RNA (exRNA) pro-inflammatory activity by RNaseA (29), are already offering promising therapeutic results.

The review summarizes the current knowledge on the mechanism of action of the RNaseA superfamily members and their contribution to innate immunity, as sentinel proteins at the extracellular space. We also briefly compared RNaseA proteins with other extracellular RNases, such as RNaseT2 family members, which are ancient RNases that are highly conserved through taxa from viruses to humans (30, 31), and bacterial RNases (19, 32) that work as defense weapons in inter-strain warfare.

## The RNase a Superfamily

The RNaseA superfamily is a vertebrate-specific gene family that has shown great divergence in a short period of time, a characteristic trait of immune-related proteins (11, 16, 33). Despite the low sequence identity between some family members (~30%), they all share a common three-dimensional structural fold and conserved motif signature (CKXXNTF). They are small secretory proteins (13–15 kDa) expressed with a short 25–27 amino acid signal peptide. The mature protein adopts an α + β kidney-shaped fold crosslinked by three to four disulfide bonds. A conserved catalytic triad formed by two His and a Lys participates in the endoribonuclease acid–base catalytic mechanism of action (8). A marked preference for cleavage of single-stranded RNA (ssRNA) is observed, with specificity for pyrimidines at the main base and a preference for purines at the secondary base site (7, 34, 35). In addition, other nucleotide-binding sites contribute to RNase-substrate specificities (36–38) and might determine the selectivity of RNases for cellular RNA. Recently, novel methodologies to identify the selective cleavage site for non-coding RNA for some family members, i.e., tRNA, have indicated their direct involvement in main cellular machinery tasks (39–41).

**Figure 1:**
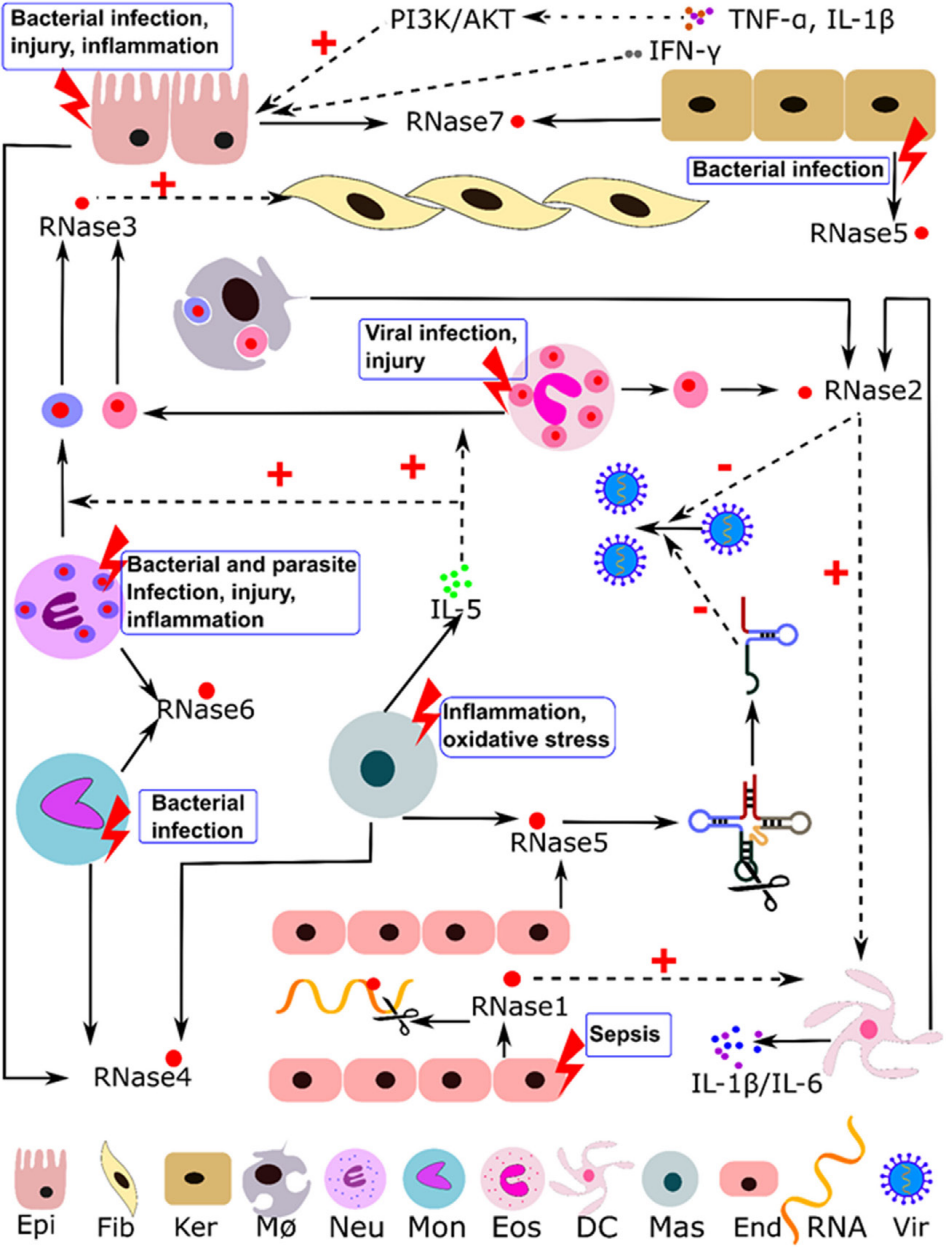
Illustration of the immuno-modulatory properties reported for human RNaseA family members. Induction stimuli, expression cell type, and regulation pathways are indicated. Abbreviations: Epi, epithelial; Fib, fibroblast; Ker, keratinocytes; MØ, macrophages; Neu, neutrophils; Mon, monocytes; Eos, eosinophils; DC, dendritic cells; Mas, mast cells; End, endothelial cells; Vir, virus; dotted line indicates regulation paths and solid line indicates expression and secretion processes.

RNaseA superfamily members are mainly expressed in innate cells and display a variety of antimicrobial and immune modulation activities. They can participate in host immune responses, working as alarmins and safeguard molecules against infection and inflammation (16, 42–45). Table 1 summarizes most of their reported activities and suggested physiological roles. Below, we describe the eight canonical family members in humans. Table 2 indicates their reported source cell types and summarizes their constitutive and induced expression patterns. Human RNase expression and response processes activated by diverse stimuli are illustrated in Figure 1.

**Table 1:** Proposed roles and reported activities for extracellular RNases.

RNases roles	Reported activities	Reference
Cellular immune regulation	– Innate cells’ activation and migration	(46–53)
	– Toll-like receptor pattern recognition and receptor activation	(46, 54, 55)
	– Hematopoiesis	(28)
	– Selective processing of non-coding RNA	(6, 40, 56–59)
	– Release of regulatory tRNA fragments	(6, 41, 58)

Tissue homeostasis, repair and remodeling	– Alarm signalling	(17, 54, 60, 61)
	– Activation and chemotaxis of fibroblasts	(51, 52, 62)
	– Activation of epithelial cells	(51, 52)
	– Cell proliferation activity	(63, 64)
	– Angiogenesis and neo-vascularization	(65, 66)
	– Wound healing activity	(67–70)
	– Autophagy induction	(71, 72)
	– Apoptosis induction	(73–75)

Clearance of extracellular RNA (exRNA)	– Clearance of cellular RNA debris following tissue injury	(29)
	– RNA scavenger activity	(76, 77)
	– Removal of blood exRNA released during hypoxia	(76)
	– Reduction of exRNA pro-inflammatory activity	(29, 78)

Epithelial barrier protection	– Antimicrobial activity at the skin and respiratory, urogenital and intestinal epithelial tracts	(43, 44, 70, 79–85)

Body fluid sterility	– Antibacterial activity	(83, 86–91)
	– Antiparasitic activity	(92–99)
	– Antiviral activity	(100–107)

**Table 2:** Expression profile and location of human secretory RNases.

Human RNase	Main expression tissues and cell types^a^	Presence in biological fluids: rank score or reported level^b^		Subcellular location^c^	Expression regulated by (in comparison with)^d^	
1	Pancreas Lung Adipose Endothelial cells Erythroblasts	Amniotic fluid: 629 Blood: 4.09e^3^ Serum: 0.5 μg/mL Synovial fluid (108) Cerebrospinal fluid (109) Urine (110)		Extracellular space Exosomes (111)	↑ Psoriatic arthritis (vs healthy in synovial fluid) ↑ Sepsis (vs normal in whole blood and monocytes) (112) ↑ Meningococcal sepsis (at 24 h vs normal at 0 h in monocytes) ↑ Leishmaniasis (vs normal) ↑ Systemic lupus erythematosus (vs normal) ↑ Interstitial cystitis (ulcer vs normal) ↓ *Francisella tularensis* novicida (vs uninfect) ↓ *Francisella tularensis* schu S4 (vs uninfect) ↓ *Mycobacterium tuberculosis* (vs none in macrophages at 48 h)	

2	Liver Lung Spleen Bone marrow Neutrophils Eosinophils Monocytes	Amniotic fluid: 1.41e^4^ Blood: 982 Cerebrospinal fluid (113) Urine (114)		Extracellular space (116) Exosomes (117) Azurophil granule lumen	↑ Septic shock (vs normal) ↑ Meningococcal sepsis	
						At 8 h, 24 h vs normal at 0 h in lymphocytes At 24 h vs normal at 0 h in blood At 0, 8, and 24 h vs normal at 0 h in monocytes At 24 h vs meningococcal sepsis at 0 h in monocytes
		Early gut lavage fluid (115)	Healthy: 163 ng/mL			
		Late gut lavage fluid (115)	Inflammatory bowel disease: 538 ng/mL Healthy: 18 ng/mL Inflammatory bowel disease: 95 ng/mL		↑ Sepsis (vs normal in whole blood, CD8, and monocytes) ↑ Tuberculosis (vs normal) ↑ Leishmaniasis (vs normal) ↑ Lyme disease at acute phase of infection (vs normal) ↑ Interstitial cystitis (ulcer vs normal) ↑ Psoriasis (at lesional skin vs normal at normal skin) ↑ Burn (vs control at early stage and middle stage) ↓ *Francisella tularensis* novicida (vs uninfect) ↓ *Mycobacterium tuberculosis* (vs none in macrophage at 48 h)	

3	Bone marrow Neutrophils Eosinophils Monocytes T cells	Amniotic fluid: 3.17e^4^ Blood: 4.51e^3^ Cerebrospinal fluid (113)		Exosomes (111, 124) Extracellular space (125) Azurophil granule lumen	↑ Meningococcal sepsis	At 24 h vs normal at 0 h in blood At 24 h vs normal at 0 h in lymphocytes At 0, 8, and 24 h vs normal at 0 h in monocytes
		Bronchoalveolar lavage fluid (118) Sputum (119)	Control: 1.7 μg/L Asthma: 2.8 μg/L Healthy: 26.1 ± 4.7 ng/mL Asthma: 142.6 ± 34.2 ng/mL		↑ Septic shock (vs normal) (112) ↑ Lyme disease at acute phase of infection (vs normal) ↑ Leishmaniasis (vs normal) ↑ Burn (vs control at early stage and middle stage)	
		Tear (120)	Control: <20 μg/L Vernal keratoconjunctivitis: 470 μg/L Atopic keratoconjunctivitis:215 μg/L Giant papillary conjunctivitis: 53 μg/L		↑ Asthma (118, 119) ↑ Inflammatory bowel disease (115) ↓ Atopic eczema (at normal skin vs normal at normal skin)	
		Early gut lavage fluid (115)	Healthy: 5 ng/mL Inflammatory bowel disease: 15 ng/mL		
		Late gut lavage fluid (115)	Healthy: 1 ng/mL		
		Plasma (121)	Control: 3.5 ± 4.1 μg/L Reactive eosinophilia with inflammation: 75.0 ± 92.3 μg/L		
		Serum (122)	Control: 5.4–9.2 μg/L Helminthiases: 46–98 μg/L Atopic dermatitis: 50 μg/L Bacterial infections: 23 μg/L Malaria: 13 μg/L		
		Nasal fluid (123)	Interstitial cystitis:10 μg/L Normal: 32.6 ± 8.1 ng/mL Chronic non-allergic sinusitis: 87.6 ± 20.8 ng/mL Perennial allergic rhinitis: 84.7 ± 24.7 ng/mL Perennial and seasonal allergy: 112.9 ± 25.6 ng/mL		

4	Liver Adipose Salivary gland Colon Endothelial cells Monocytes B cells T cells	Blood: 9.27e^3^ Cerebrospinal fluid (113)	Extracellular space Exosomes (126)		↑ Sepsis (vs normal in whole blood) ↓ Visceral Leishmaniasis (vs normal) ↓ Psoriasis (at lesional skin vs normal at normal skin)	

5	Liver Endothelial cells Spinal cord neurons T cells Mast cells	Amniotic fluid: 2.60e^4^ Blood: 7.93e^3^ Plasma: 96–478 ng/ml (127) Bronchoalveolar lavage fluid (128)		Exosomes (111) Extracellular space Growth cone Basal lamina (131) Angiogenin–RNase inhibitor complex (132)	↑ *Enterococcus faecalis* (vs none at 5 days) ↑ *Mycobacterium tuberculosis* (vs none in macrophage at 48 h) ↑ Meningococcal sepsis (at 24 h vs normal at 0 h in blood) ↑ Asthma (128) ↑ Inflammatory bowel disease (129 ↓ *Francisella tularensis* novicida (vs uninfect))	
		Serum (129)	Healthy: 394.6 ± 137.6 ng/mL	Neuronal cell body	↓ *Francisella tularensis* schu S4 (vs uninfect)	
		Cerebrospinal fluid (130)	Ulcerative colitis: 526.5 ± 224.1 ng/mL Crohn’s disease: 508.8 ± 228.5 ng/mL Crohn’s disease: 508.8 ± 228.5 ng/mL Control: 6.197 ± 1.987 ng/ml Amyotrophic lateral sclerosis: 5.582 ± 1.754 ng/mL	Nucleus (133) Nucleolus (134, 135) Cytoplasmic vesicle RNA stress granules (58)	↑ Psoriasis	Lesional vs non-lesional psoriasis Lesional vs normal

6	Lung Heart Placenta Kidney Monocytes Neutrophils	Amniotic fluid: 1.49e^4^ Blood: 1.87e^3^		Exosomes Extracellular space Cytoplasmic vesicle	↑ Ulcerative colitis ↑ Crohn’s disease ↑ Interstitial cystitis (ulcer vs normal) ↑ Tuberculosis (vs normal) ↑ Juvenile dermatomyositis (vs normal) ↑ Interstitial fibrosis and inflammation (vs normal) ↑ Sepsis (vs normal in whole blood) ↑ Periodontitis (vs normal)	
					Meningococcal sepsis	↑ At 8 h, 24 h vs normal at 0 h in lymphocytes ↓ At 0 h vs normal at 0 h in monocytes
					↓ *Francisella tularensis* novicida (vs uninfect) ↓ *Francisella tularensis* schu S4 (vs uninfect) ↓ *Mycobacterium tuberculosis* (vs none in dendritic and macrophage cell at 18 and 48 h) ↓ Newcastle disease virus (at 14, 16, and 18 h vs none at 0 h) ↓ Psoriasis (vs normal) ↓ Septic shock (vs normal) ↓ Burn (middle stage vs control)	

7	Epithelial tissues Liver Kidney Skeletal muscle Keratinocytes (89, 136) Basal cells (70)	Amniotic fluid: 2.69e^3^ Blood: 3.22e^4^ Skin washing fluids (44)	Forehead: 0.93 ng/cm^2^ Nose: 2.7 ng/cm^2^ Arm: 0.94–4.9 ng/cm^2^ Palm: 2.0 ng/cm^2^ Finger: 1.5 ng/cm^2^ Hand back: 2.0 ng/cm^2^ Neck: 3.1 ng/cm^2^ Calf: 3.4 ng/cm^2^	Exosomes (111, 137, 138) Extracellular space (43, 84) Cytoplasm	↑ Sepsis (vs normal) (112) ↑ Psoriasis (lesional vs normal) ↑ Acute pyelonephritis (84) ↑ Atopic dermatitis (69) ↑ Chronic anal fistula (139) ↑ Nontypeable *Haemophilus influenzae* (70) ↑ *Pseudomonas aeruginosa* (89) ↑ *Staphylococcus aureus* (89) ↑ *E. coli* (89) ↑ *Streptococcus pyogenes* (89) ↑ *Mycobacterium tuberculosis* (140) ↓ Atopic eczema (at skin lesion and normal skin vs normal at normal skin)	

8	Placenta Spleen (141) Lung (141) Testis (141)			Extracellular space		

*^a^Summary from Uniprot (www.uniprot.org/uniprot/), Genevisible (https://genevisible.com/search), and Human Expression Atlas (https://www.ebi.ac.uk/gxa/home/)*.

*^b^Summary from Bgee (https://bgee.org/) (Rank scores of expression calls are normalized across genes, conditions, and species. Low score means that the gene is highly expressed in the condition. Max rank score in all species: 4.10e^4^. Min rank score varies across species)*.

*^c^Summary from Uniprot (www.uniprot.org/uniprot/) and Exocarta (www.exocarta.org/)*.

*^d^Summary from Human Expression Atlas (https://www.ebi.ac.uk/gxa/home/). Only inflammatory, injury, and infection-related conditions are included (up and downregulation is indicated by arrows)*.

### hRNase1

hRNase1 is the human homolog of the family reference protein, bovine pancreatic RNaseA. A comparative evolutionary analysis indicates a divergent role for non-ruminant RNase1 family members that are unrelated to digestion (18, 142). Expression of human RNase1 was detected in almost all tissues (143). In particular, human RNase1 is abundantly expressed by endothelial cells (144, 145), and its presence in blood can modulate the content of exRNA (18, 77). An interesting hypothesis attributes an RNA scavenger role to hRNase1 (77). Supporting this proposal, Raines and co-workers (18) highlight the different optimal conditions for catalysis between ruminant and non-ruminant RNases. Whereas bovine pancreatic RNase shows a slightly acidic optimum pH for catalysis, adapted for working in the digestive tract (4), the observed optimum neutral pH for non-digestive RNases allows their enhanced activity in biological fluids such as blood (18). Likewise, the enhanced capacity to cleave double-stranded RNA (dsRNA) by non-digestive RNases (10, 18) might facilitate the removal of heterogeneous RNAs circulating in blood (18). Interestingly, clearance of exRNA would mitigate its pro-inflammatory activity (76, 77). An excess of exRNA is released in hypoxic conditions, and hRNase1 administration was reported to provide cardiac protection in a mouse model (76). Administration of the protein *in vivo* reduces the release of pro-inflammatory cytokines and provides a multiple-organ protection in mice (29).

### hRNase2

hRNase2, together with hRNase3, is one of the main secretory proteins stored within the eosinophil secondary granules (107, 146). This eosinophil protein is partly responsible for eosinophil-induced neurotoxicity, and it is referred to as the eosinophil-derived neurotoxin (EDN), hereafter. In addition, hRNase2/EDN is expressed in other blood cell types (Table 2) such as monocytes and dendritic cells (DCs) (107). Expression of the protein is induced by viral infection (100, 107, 147), and it displays a broad antiviral activity, in particular against ssRNA viruses (104, 107). The protein inhibits the replication of respiratory syncytial (RSV), human immunodeficiency virus (HIV)-1, and hepatitis B DNA virus (100, 102, 103). RNase2 has high catalytic activity, which together with its unique protein structural features (106), is essential for its antiviral action (104). Eosinophil recruitment and degranulation is activated during RSV infection (147), and hRNase2 is a clinical marker for RSV bronchiolitis (148). Interestingly, Weller and co-workers observed the induced release of eosinophil RNases by cytokines such as eosinophil chemotactic proteins (eotaxins CCL11 and CCL24) and through the P13K/MAPK pathway in humans and mice (149). Eosinophil activation by virus infection can be mediated by the toll-like receptor (TLR)7–MyD88 signaling pathway. Likewise, specific expression of the eosinophil RNases following RSV infection in mice is reported to be mediated by the TLR7–MyD88 pathway (55). The TLR7 receptor can be activated by ssRNA, causing the eosinophil RNase to act on viral RNA to enhance the host response against the infection. In addition, viral infection of the respiratory tract is frequently accompanied by lung inflammation and hRNase2 expression. Secondarily, local eosinophil degranulation correlates with tissue damage with eosinophil degranulation activated by tissue injury (Figure 1) and the released RNases contributing to the tissue remodeling process (149). Indeed, hRNase2 immuno-regulatory properties can promote leukocyte activation, maturation, and chemotaxis (107). In particular, hRNase2 can contribute to host immunity through interactions with DCs (46, 47). The activation of DCs can be mediated by the TLR2–MyD88 pathway, and hRNase2 is classified as an alarmin (60). Direct protein binding to TLR may occur (107) taking into account the striking shape similarity between the TLR receptor and the RNase inhibitor (RI), both of which have leucine-rich repeat (LRR) domains (150). Human RNase2 and mouse RNases2 are also both expressed in macrophages (Table 2) (48, 107). Of note, the mouse eosinophil-associated RNase Ear11 works as a macrophage chemoattractant, although, in this case, the process is not directly mediated by TLR2 activation (48, 107). Because TLRs can interact with ssRNA, we can contemplate a scenario in which RNA pieces bound to RNase2, or, alternatively, the direct action of RNase cleavage products, trigger the TLR signaling cascade. As TLRs can discriminate between self and non-self molecules, cleavage of viral RNA could contribute to the host defense response. Thereby, immune regulation and antimicrobial functions would work cooperatively (151). Eosinophil degranulation during infection and inflammation can contribute locally to both eradicate the infection focus and palliate tissue injury.

### hRNase3

hRNase3 is the other eosinophil RNase abundant in the secondary secretory granule, where both RNase2 and 3 together account for about one-third of the total protein content (152). The two human eosinophil RNases share 70% amino acid identity. RNases 2 and 3 emerged from a gene duplication event about 50 million years ago and underwent a divergence process at an extremely rapid rate of evolution (12). During the drift of RNase3 from a common RNase2/3 ancestor, the protein acquired much higher cationicity (pI > 10); therefore, it is called eosinophil cationic protein (ECP). Abundant surface-exposed Arg residues facilitate binding of the protein to bacterial cell membranes and subsequent destabilization through a carpet-like mechanism characteristic of many host defense antimicrobial proteins and peptides (AMPs) (153). RNase3 shares neurotoxic and antiviral activities with RNase2 (105) but it has unique bactericidal properties (86). In particular, hRNase3, together with its high cationicity, has an aggregation-prone region that promotes the protein self-aggregation and mediates the agglutination of bacterial cells (154). Bacterial cell agglutination is further enhanced by high binding affinity of the protein to anionic lipopolysaccharides in the Gram-negative bacterial wall (155). RNase3 release is induced both by infection and inflammation, and several immuno-modulatory activities have been described. The levels of protein circulating in biological fluids (Table 2) correlate with eosinophil degranulation, and it is currently used as a routine clinical marker for the diagnosis and monitoring of inflammatory disorders, such as asthma (51, 52). Airway inflammation is closely associated with eosinophil degranulation and local tissue damage follows the deposition of eosinophil secondary granule protein (156, 157). The levels of this protein are also associated with damaged airway epithelia (52). Further, skin ulceration follows eosinophil infiltration, and local protein deposits harm epithelial cells (121, 158, 159). Fortunately, the detrimental side effects of the protein tissue deposits are followed by remodeling processes. RNase3 remodeling activity is partly mediated by the upregulation of the insulin growth factor-1 receptor on epithelial cells. In addition, hRNase3 activation and chemotaxis of fibroblasts can contribute to tissue repair (49, 62, 160). Nevertheless, fibroblast activation can also lead to airway fibrosis, as observed during chronic eosinophil inflammation in asthma (49). Interestingly, a population study identified a natural genotype variant of hRNase3 (ECP97Arg) with enhanced cytotoxicity that was linked to a higher frequency of fibrosis (161). Despite the higher antimicrobial activity of the Arg97 variant, genetic selection toward a less toxic protein must have taken place in a chronic parasite infection in endemic areas of Asia to reduce the incidence of liver fibrosis (162). The Arg to Thr substitution at position 97 of hRNase3 results in a new potential N-glycosylation site at a nearby Asn residue (163). Additional glycosylation at this site blocks the cationic domain that participates in the protein antimicrobial activity (164). Similarly, a correlation between hRNase3 polymorphisms and cerebral malaria susceptibility was observed (165, 166).

Together with direct hRNase3 action on pathogens and host tissues, a series of immune-modulating activities are observed (51, 52). Eotaxin attracts eosinophils to the area of inflammation, i.e., lung in asthma, nasal mucosa in allergic rhinitis, skin in dermatitis, or gut epithelia in intestinal bowel diseases. Eosinophil degranulation is activated by IL-5, leukotriene B4 (LTB4), platelet activating factor (PAF) (52), or the P13K/MAPK pathway (149). Early experimental assays also showed mast cell activation by hRNase3 and the induction of histamine release (50). In turn, mast cells produce and secrete IL-5, PAF, and LTB4, which enhance hRNase3 release from eosinophils. Moreover, hRNase3 induces the synthesis of prostaglandin D2 by mast cells, which then acts as an eosinophil chemoattractant. This process suggests cross talk between mast cells and eosinophils, with RNase able to promote positive feedback (52). Other leukocyte cells such as neutrophils can also express hRNase3 (Table 2). Interestingly, free granules released by both eosinophils and neutrophils maintain their autonomy and functionality (167) and selectively secrete RNases upon cytokine induction (168). Moreover, the extracellular granules can also be engulfed by macrophages (Figure 1) and contribute to the immune response.

### hRNase4

hRNase4 is one of the oldest representative RNase A family members within mammals, showing a static evolution history in comparison with the other family counterparts (33). RNase 4, together with RNase 5, shares some conserved structural features with non-mammalian vertebrates RNases, such as the first pyroglutamic N-terminal residue (169). Moreover, there are no reported glycosylation forms and no recognition sites for N-glycosylation (169). RNase4 retains the highest inter-species homology, close to 90%, within the family members (33, 170). Nearly ubiquitous distribution suggests a housekeeping role for this protein. The presence of hRNase4 transcripts was detected in most human tissues (143, 171) and was found particularly abundant in the liver (143) and lungs (169). Interestingly, cytoplasmic granules of monocytes also express this RNase (172). Divergence and diversification events within the oldest mammals suggest a strong evolutionary pressure that may respond to host adaptation to an ever-changing pathogen environment. In this scenario, duplication of the RNase4 gene in ancestral mammals may have led to other RNaseA family lineages that acquired a host defense function (33). There is a controversy about the physiological role of the protein. On one side, its conservation among species suggests strong evolutionary pressure to maintain an essential role. Cleaning-up of cellular RNA was first proposed (173). However, the enzyme showed enhanced preference for uridine at the main base binding site (169, 173, 174), suggesting strong selectivity for RNA recognition (169). A structural analysis highlighted particular structural features at the main base binding pocket that determine the protein enhanced preference for uridine over cytidine in comparison to other family members (174, 175). Although considerable work has been undertaken to interpret RNase4 substrate specificity, there are few reports on the biological properties of the protein. Interestingly, RNase4 was identified, along with RNase5, among the soluble factors secreted by T cells showing anti-HIV activity (176).

### hRNase5

hRNase5 is considered the most ancient RNaseA family member, and it shares many structural features with non-mammalian vertebrate RNases. It is unique within the family in presenting six paired cysteine residues instead of eight. In addition, it shows rather atypical enzymatic properties, with very low catalytic efficiency for ssRNA but selective cleavage for some non-coding RNA (41, 177, 178). Its expression was detected in many adult and embryonic somatic cells (179). It was also reported in a variety of innate cells, ranging from diverse blood cell types to intestinal and skin epithelial cells (Table 2) (28, 67, 129, 180). Further expression of this protein is increased during inflammation (129, 181). RNase5 promotes angiogenesis and is, therefore, termed angiogenin (65). The purified protein was reported to display other activities, such as antimicrobial action (67, 88) and some immuno-regulatory properties (88, 182), in addition to the induction of vascularization. In particular, the protein inhibits neutrophil degranulation, a process that might induce an anti-inflammatory effect during immune response (183, 184). Interestingly, this degranulation inhibitory action was mimicked by a short tryptic peptide (183), indicating a protein activity unrelated to its enzymatic function. On the other hand, angiogenesis relies on hRNase5 catalytic activity and is inhibited by the RI (185). Distinct immuno-regulatory activities were observed to be dependant on the action of the protein on non-coding RNA, i.e., cleavage of tRNA and upregulation of rRNA. Upon nuclear translocation hRNase5 can stimulate the proliferation of several cell types, such as endothelial cells (66), by regulating rRNA transcription (63, 186). In addition, angiogenesis and cell proliferation are mediated by the activation of cellular signaling kinases such as the ERK1/2 (179, 187). In addition, hRNase5 expression is activated in response to cellular stress and promotes the formation of stress granules. The protein generates stress-induced tRNA fragments (tRFs) (tiRNA) (6, 56, 58). In turn, the tiRNA fragments can impede the formation of the translation initiation factor complex, thereby inhibiting translation (188). Accumulation of tiRNA activates the cell response to oxidative stress (59). The release of tRFs associated with hRNase5 activity is also a characteristic feature linked to endoplasmic reticulum (ER) stress, a condition that can be triggered by the accumulation of unfolded/misfolded proteins in the ER lumen (57). Likewise, hRNase5 induces the release of tiRNA by stem cells and activates hematopoietic cell regeneration (28). Generation of tRFs is also observed upon viral infection (189). Using deep sequencing methodologies, the enzyme cleavage target sites are being identified (5, 6, 39). Two tRNA halves can be produced by a unique enzymatic cut at the anticodon loop (5, 6, 58, 190). In addition, Li and co-workers observed a unique cleavage by hRNase5 at the tRNA TΨC loop (39); the resulting 3′-tRNA fragment was complementary to an endogenous human genome sequence, and the fragment was found to downregulate retroviral expression by RNA interference (39). However, potential therapeutic applications for hRNase5, such as hematopoiesis regeneration or antiviral activity (28), should be viewed with caution, considering its protein pro-tumoural properties (65). In particular, upregulation of rRNA transcription has been related to cancer cell proliferation (63, 64). A recent work correlated elevated hRNase5 expression in some tumor cell lines with the promotion of cell proliferation and development of malignant cancer (64). In addition, elevated levels of tiRNA were also observed in some cancers (190) with tRFs inducing tumorigenesis (188).

### hRNase6

hRNase6, also named hRNasek6, was identified for the first time by Helen Rosenberg and Kimberly Dyer as a human ortholog of bovine kidney RNasek2 (191). The first tissue screening study to define hRNase6 expression patterns revealed a nearly ubiquitous distribution, including monocytes and neutrophils (191). The expression of this protein is upregulated following genitourinary tract bacterial infections (83) and high antimicrobial activity against Gram-positive and Gram-negative species was observed (83, 90). Very recently, hRNase6 antiviral activity was reported. Interestingly, the authors observed the protein downregulation in Th17 polarized cells upon HIV infection (192).

### hRNase7

hRNase7 is probably the best studied example of an RNase that can work as a tissue safeguard sentinel (Table 1). It is also one of the most abundant antimicrobial proteins purified from skin (44, 79). RNase7 is secreted by a variety of epithelial cells (Table 2) and mostly contributes to urinary tract sterility and epidermis protection (43, 80–82). Together with high antimicrobial activity against a variety of infective microorganisms (82, 87, 89, 193), some immuno-modulatory properties were reported. RNase7 expression is upregulated during kidney infection (84). Expression is also selectively induced by inflammation signaling molecules, such as IL-1β and IFN-γ (89) or the PI3K/AKT pathway (194). The PI3K/AKT signaling pathway can modulate the innate immune response during inflammation and prevent sepsis (195, 196). Interestingly, expression of this protein in the urinary tract can be upregulated by insulin through the PI3K/AKT pathway (197). Spencer and co-workers correlated expression with increased susceptibility to infection of diabetic patients (197). Indeed, insulin induces the secretion of granulocyte content and impairment of the expression of other AMPs, which are also associated with diabetes (198). Of note, the regulation of protein expression by the P13K/AKT pathway is also observed with eosinophil-associated RNases (EARs) (149). On the other hand, RNase7 is abundantly secreted by keratinocytes (43, 44, 79) and can contribute to wound healing and tissue repair (67, 68). Protein overexpression in skin can be induced by inflammation and infection diseases (69, 81). Interestingly, protease degradation of the RI at the stratum corneum can activate hRNase7 for skin barrier protection (67). Finally, expression of the protein was recently reported to be induced in basal cells of damaged airway epithelia, reinforcing the idea of a protective role for this protein following tissue injury (Table 1) (70). A very recent study showed that this protein directly stimulated plasmocytoid DCs following tissue damage and infection, and the authors of the report proposed to classify hRNase7 as an alarmin (54). Interestingly, the immuno-modulatory activity of the RNase correlates with binding to self-DNA and activation of TLR9 receptors. The authors suggest that following tissue damage, hRNase7 detects the host DNA released by dying cells and activates the host response (54).

### hRNase8

hRNase8 is the last identified and least well-characterized canonical member of the RNaseA superfamily. It was first uniquely identified in the placenta (199). Wide spectrum antimicrobial activity was observed for this protein (91), suggesting a role in amniotic fluid protection against infection. Indeed, hRNase7, the closest homolog to hRNase8 in the RNaseA family, was found recently among AMPs expressed in prenatal skin, suggesting it may contribute to amniotic cavity sterility (200). However, despite sharing a high sequence identity with hRNase7, hRNase8 shows highly reduced catalytic activity (199). A particular cysteine location within its primary sequence indicates a unique disulfide bonding among the family members. An evolutionary analysis in primates revealed a sequential cysteine gain-and-loss process, representing an unusual example of disulfide bond reshuffling (201). In addition, the protein shows an elevated rate of incorporation of non-silent mutations in its primary structure (202) suggesting functional divergence toward a distinct physiological role (141). Moreover, a unique extension at the hRNase8 N-terminus may indicate that the protein is not undergoing the secretion process shared by all other canonical members of the family (141). Recent evidence of hRNase8 gene expression in other additional tissues, such as the lung, liver, and testes (141), together with controversial reports on its recombinant protein antimicrobial activity (91, 199), urges a reconsideration of the function of this protein.

### hRNases 9–13

The RNaseA superfamily was lately expanded with the discovery of several novel mammalian members (11, 203–205). The newcomers share just 15–30% identity with the eight “canonical RNases,” and they are associated mainly with male reproductive functions. Some of them, like hRNase9, are endowed with bactericidal activity and are expressed in the epididymis, with evidence of an association with sperm maturation (33, 206). The primary structure of these proteins resembles ancestral RNases, sharing the three most conserved disulfide bonds and a secretion peptide, but not the N-terminus region of mature proteins. In addition, the non-canonical RNases do not include the family signature (CKXXNTF) or the catalytic triad and their biological properties do not seem to require enzymatic activity (207).

### Other Vertebrate RNaseA Family Members

The RNaseA superfamily is one of the most extensively studied gene family, since the pioneering studies on molecular evolution (208). As a vertebrate-specific family, it is an excellent working model and a deep analysis has been carried out to understand the main driving force toward a defined function in mammals (11, 33, 209, 210). Further, we will briefly comment on the non-human family members present in the extracellular compartment and displaying immune modulation properties. In particular, bovine proteins are the best characterized, being the RNaseA the bovine counterpart of human RNase1. RNaseA, the bovine pancreatic RNase1, is expressed in large amounts in the pancreas and participates in RNA degradation in the digestive tract (3).

### Bovine Seminal RNase (BS-RNase)

Bovine seminal RNase is a close homolog to bovine pancreatic RNase that is solely present in seminal fluid (211), where it plays an immune-protective role (182). BS-RNase, despite sharing an 80% amino acid identity with the pancreatic RNaseA, is the only family member present *in vivo* as a homodimer. Owing to its natural dimeric form, BS-RNase can elude the blockage of the cytosolic RI in case of cellular internalization following endocytosis. The high cytotoxicity of the protein (212) is attributed to the degradation of cellular RNA (213). In addition, the dimerization of BS-RNase and RNaseA constructs correlates with an enhanced catalytic activity and the ability to cleave dsRNA (214–216). Degradation of dsRNA by BS-RNase is induced by IFN (217, 218) and the protein can inhibit HIV-1 replication in leukemia cells (219). Interestingly, engineered quaternary structures of RNaseA can not only mimic BS-RNase enzymatic cleavage of dsRNA, but also some of its biological properties (220, 221). The seminal RNase also differs from its pancreatic homolog by its ability to inhibit the proliferation of cancer cells mediated by autophagy induction (72). BS-RNase also inhibits the proliferation of T-lymphocytes (222, 223) and can downregulate the T cell IL-2 receptor expression (222). However, the immune-protective mechanism of BS-RNase in the seminal fluid remains unknown (182).

### Bovine Milk RNases

Bovine milk RNases are another group of secretory RNases that mediate an extracellular protective role. Two proteins with RNase activity homologous to human RNases 4 and 5 were identified in bovine milk (224, 225). Both RNases were quantified in bovine milk at μM concentrations and reported to display some antimicrobial activity (225). Bovine milk RNases can participate in the host response against infection both by direct antimicrobial action and immune response activation (182). A pro-inflammatory activity is observed in epithelial cells, which is mediated by nucleic acids (53, 226). Both RNases can bind nucleic acids, and milk RNase5 induces cytokine release in leukocytes (53). Recognition of foreign pathogen nucleic acids may facilitate the activation of pattern recognition receptors and promote a pro-inflammatory response (226). Interestingly, the RNase immuno-stimulatory activity is also dependent on the protein catalytic activity (226).

### Rodent RNases

Rodent RNases are another well characterized group that can help us to outline the RNaseA family involvement in the host immune response (11). Lineages of RNases 1, 2, 3, and 5 are identified in rat and mouse genomes, presenting an unusual expansion rate (11, 207, 227–229). However, no orthologs of RNases 7 and 8 have been found (11, 33). Particularly, a striking diversity of RNases 2 and 3 counterparts is observed in mice and rats. Two orthologs of the eosinophil RNases lineage were first discovered in 1996 and named EARs (230). Subsequently, up to 13 new eosinophil murine members were identified (231). The phylogenetic analysis of the distinct EAR rodent gene clusters revealed a rapid gene duplication and selection process that resulted in high diversification, a characteristic pattern of host defense protein lineages (232). Many of these EAR proteins, despite their nomenclature, are not solely secreted by eosinophil granules but can also be expressed by other cell types. For example, mEAR11 is expressed in somatic tissues, such as lungs, liver, or spleen, along with macrophages. The protein expression is induced in response to Th2 cytokines and it acts as a potent leukocyte chemoattractant (48). The immune regulation of eosinophil release of granule proteins in mice has been thoroughly studied by Weller and colleagues (149, 168). EARs similar to their human counterparts actively contribute to the host defense and tissue repair and remodeling. However, significant differences are observed in the regulation mechanism of eosinophil degranulation, limiting the use of laboratory animal models in the study of human eosinophil-associated diseases (233–235). Nonetheless, experimental studies in mice corroborate the autonomy of eosinophil cell-free granules and their activation by a common CCR3-mediated signaling pathway (149, 168). EARs can also provide immune protection against virus infection *in vivo* in a mice model, where the eosinophil activation and virus clearance is mediated by a TLR7-signaling pathway (55).

In addition, several homologs to human RNase5 endowed with antimicrobial properties were identified in mouse intestinal epithelium (88). In particular, the upregulation of the mouse RNase Ang4 by commensal bacteria suggests a role for this protein in the gut and systemic innate immunity, where it can establish a host defense barrier against infection (88).

Rosenberg and colleagues went further down the evolution scale and characterized avian and reptilian RNases to deepen the understanding of the role of vertebrate RNaseA family in host immunity (13, 16, 236). As mentioned previously, non-mammalian RNases are evolutionarily closely related to mammalian RNase5 members (11). Two leukocyte-associated homologs were identified in chicken, RNases A1 and A2, the last one displaying both angiogenic and bactericidal properties (13). On the other hand, the *Iguana* RNase is catalytically active but devoid of antimicrobial activity (236).

### Frog RNases

Frog RNases are secreted by oocytes and early embryos and might protect the eggs against infection (237–241). The observed anti-proliferative properties of the RNases from early vertebrates have attracted the interest of pharmaceutical companies since their discovery (242, 243). *Rana pipiens* RNase, named Onconase^®^ (ONC) after its anti-tumoural activity, is currently on phase III clinical trial. The anti-proliferative action of ONC on cancer cells is mediated by induction of the autophagy pathway (71), as reported for BS-RNase (72). Recently, its cytotoxicity was enhanced by promoting its dimerization (244). The anti-tumoural activity of ONC has also been attributed to its action on microRNA (miRNA) precursors (40). On the other hand, the frog RNase inhibits the replication of HIV-1 through directly targeting the viral RNA and host cellular tRNA (245, 246). A specific excision on host Lys-tRNA inhibits the virion replication (101). A common tRNA targeting mechanism might be shared with the oldest mammalian RNases. Moreover, a similarity between the structural fold of tRNA and miRNAs that are targeted by the frog RNase suggests that the RNA recognition and cleavage requires specific primary and secondary structures (40).

Finally, identification and characterization of fish RNases completes the overall picture of the vertebrate RNaseA family (15, 247, 248). RNases identified in zebrafish (ZF-RNases) shared the bactericidal, angiogenic and reduced catalytic properties of hRNase5 (247). Catalytic activity for ZF-RNase5 was required for angiogenesis but not for antimicrobial action (247). Interestingly, ZF-RNases 1 to 3 can activate the ERK1/2 kinase pathway similar to hRNase5 (249). Recently, the expression of several RNase2 and RNase3 paralogs in the pond-cultured blunt snout bream fish induced by bacterial infection was reported (250).

## Other Extracellular RNases

### RNases T2: A Family of Ancient Extracellular RNases

RNaseT2 family, in contrast with the RNaseA family, comprises a group of proteins conserved from virus to humans, suggesting a shared preserved function (31). The biological properties of human RNaseT2 have been extensively studied. The RNase that works as a signaling molecule and is secreted by damaged tissues has being classified as an alarmin (61). RNaseT2 is stored in the lysosomal compartment and contributes to the clearance of cellular macromolecular debris. Its secretion can be induced by oxidative stress and it participates in the regulation of immune response. RNaseT2 is proposed to work as an RNA scavenger in the extracellular compartment (31). Moreover, the human RNaseT2 shows macrophage chemotaxis (30) and tissue remodeling activities *in vitro*. Similarly, the RNaseT2 secreted by the eggs of the parasite *Schistosoma mansoni*, also named Omega-1, can induce the release of pro-inflammatory cytokines by macrophages during infection (251). Omega-1 can be internalized into DCs and regulates their programming pathway by the RNase-mediated cleavage of rRNAs and mRNAs and subsequent impairment of protein synthesis. Another RNaseT2 family member that has been well characterized is the yeast RNaseT2, named Rny1, which is stored in cell vacuoles, similar to the storage of other RNaseT2 members in lysosomes, and shows a selective tRNA cleavage under oxidative stress equivalent to the activity reported for hRNase5 (56). In addition, yeast RNaseT2 combines its enzymatic action with other non-catalytic properties such as binding to regulatory proteins and the destabilization of lysosomal membrane, a mechanism that can trigger the programmed cell death (56, 252). Overall, we observed common properties between the RNaseA and the RNaseT2 family members, e.g., release of stress-induced tiRNA, leukocyte activation, or exRNA scavenging (30, 31, 56, 250).

### Plant Self-Incompatibility RNases (S-RNases)

Plant S-RNases prevent self-fertilization and avoid inbreeding. S-RNases exert cytotoxicity against the growing pollen tube by targeting rRNA (253, 254). Each plant is endowed with specific recognition patterns that can block the RNase activity of all the S-RNases except its own, ensuring the degradation of pollen grains corresponding to its haplotype (255). Interestingly, S-RNases exhibit a specific catalytic activity on tRNA when the plants are exposed to stress (256).

### Bacterial RNases as Inter-Strain Competition Toxins

Going further down the evolutionary scale, we can find a wide variety of bacterial RNases that participate in the bacterial defense against external threats, e.g., presence of a competing bacterial species, viral infection, or the defense response of the infected host cell. Bacterial RNases can work as powerful toxins selectively targeting coding and non-coding RNAs (257–259). Among the non-coding RNAs, the specific cleavage of tRNAs is a conserved regulatory mechanism shared from bacterial to mammalian cells (56). Stress-induced tRNA cleavage is reported for the *Escherichia coli* endoribonuclease Prrc in response to bacteriophage infection (260). Colicins are another group of *E. coli* cytotoxic tRNases that block the protein synthesis machinery as a defense mechanism against other microbial competitors (261). Interestingly, comparison among the bacterial RNases suggest an evolutionary convergence to acquire structural features that enable the targeting of the tRNA anticodon loop (261). In simple eukaryotes, such as the protozoa *Tetrahymena*, and the budding yeast *Saccharomyces cerevisiae*, the release of specific tRFs during starvation is also reported (256, 262). We can establish a parallelism between the release of stress-induced tiRNA by prokaryotes, primitive eukaryotes, and human RNase5 (6), as a mechanism to downregulate protein synthesis. An intriguing question arises: have the host defense mechanisms of vertebrate RNases evolved from the ancestral prokaryotic inter-strain competition processes? Although the RNaseA superfamily is vertebrate-specific, the recent report of the structure of a bacterial RNase involved in inter-strain competition highlights a shared protein scaffold shaped for RNA recognition (32). However, the lack of sequence identity between bacterial and vertebrate RNases and the absence of any putative invertebrate intermediate suggests a convergent evolution (32, 263). Thus, the origin of the RNaseA superfamily remains unknown. In contrast, the RNaseT2 family conserves its ancestral lineage from prokaryotes to humans (56).

Similarities between unicellular self-defense and mammalian innate immune mechanisms can provide novel strategies to boost our own immune response. For example, macrophage immune regulation by *Bacillus* RNase (binase) can trigger the host cell anti-tumor response (19) and the RNaseT2 of the *Schistosoma* parasite can modulate the host response (250) and prevent the outbreak of autoimmune diseases or diabetes (264, 265). Understanding the uniqueness of RNases and their specificity for cellular RNAs will lead to the development of novel therapeutics.

## RNase Traffic in the Extracellular Space

Extracellular RNases are released as secretory proteins by diverse pathways into the extracellular compartment. Recent advances in histochemical and cell analytical methodologies have unveiled the structural and functional complexity of the extracellular space. A rich world of secretory storage granules, transport vesicles, and intracellular vacuoles ensures that the organism is fit to respond to external stimuli.

### Compartmentalization

Compartmentalization of RNA and RNases is an important regulatory mechanism (266). RNases packed within secretory granules will be selectively released upon action by diverse stimuli (see Figure 1). In particular, eosinophil degranulation has been thoroughly investigated and several secretory mechanisms have been described (267). Intracellular granules can undergo piecemeal degranulation, whereby small packets of derived vesicles are mobilized toward the cell surface for secretion (231). Alternatively, the cell storage granules can be freed as independent entities. Free extracellular eosinophil granules can actively release their content upon cytokine activation (167). Weller and co-workers have extensively characterized the signaling pathways that mediate the release of RNases by the free extracellular eosinophil granules in humans and mice (149, 168). Free eosinophil granules can be internalized by other innate cells, such as macrophages (Figure 1) and thereby participate in the regulatory pathways of the recipient cell. Extracellular RNases can also find a way back into cells through the endosomal pathway (240, 268). Fortunately, the cytosolic compartment of cells is protected from the potential toxicity of RNaseA superfamily members by the action of the RI, which constitutes about 0.1% of the total protein content in the cytosol of mammalian cells (269). The RI is expressed in all studied human tissues (143) and binds with an extremely high affinity to mammalian RNaseA family members (in the fM range) (270, 271). Interestingly, Raines and co-workers (270) identified the avian and reptilian counterparts of mammalian RI but no equivalent protein was detected in amphibians and fish, suggesting a specific role for RI in higher order vertebrates (270). Recent RI-knockout experiments confirmed the protective action of RI toward cytosolic RNA against endocytosed RNases (272). The inhibitor structure adopts a horseshoe conformation composed of LRRs and exposed free cysteine residues. The inhibitor is functional in its fully reduced state and is extremely sensitive to cellular oxidative stress. RI inactivation by partial oxidation can work as a mechanism to switch on the RNase-mediated degradation of cellular RNA under stress conditions (56, 270). For example, RI participates in the regulation of hRNase5 subcellular localization during stress conditions. Under stress, the cytosolic hRNase5 is liberated from the RI complex, whereas the nuclear protein is bound to the inhibitor, thereby downregulating cell growth (272). Interestingly, the hRNase5 evasion of the cytosolic RI and migration to the nucleolus is also mediated by phosphorylation (273). RI can also participate in the regulation of RNases expressed at the epidermis (67). The secreted RNases in the skin provide a protective barrier against invading pathogens. Degradation of RI by proteases at the stratum corneum can liberate the RNases’ antimicrobial action during infection (67, 274). Regulation of RNase activity by RI in the urinary tract has also been proposed by Spencer and co-workers (275).

### Intercellular Communication

Nowadays, novel methodologies have led to better understanding of the functions of the extracellular compartment and have proposed previously undescribed roles for secretory proteins. RNases secreted by diverse stimuli (Figure 1) can participate in intercellular communication in an organism (56). Of note, some RNaseA family members have been detected within extracellular vesicles (117, 276, 277) (Table 2) and selective RNA packaging into the vesicles has been observed (278). This brings us back to the pioneering biochemical work on the pancreatic RNaseA family (3). The identification of the angiogenic activity of hRNase5 and the suspicion that angiogenic factors might contain RNA (279) hinted that exRNA might work as an intercellular communicator (1). Novel sequencing methodologies confirmed hRNase5 selective cleavage of non-coding RNAs and the involvement of the released products in immune regulation pathways (57). Other pancreatic RNase family members may also have evolved to acquire a non-digestive role and may contribute to the regulation of the circulating exRNA content in blood (18, 280). James Lee proposed that during vertebrate evolution the mechanism of action of granulocyte proteins might have evolved from mere localized action to an organized systemic response mechanism. The increase in size and complexity of multicellular organisms is accompanied by long distance stress signaling processes. In this context, secretory RNases originally recruited at the damaged tissue site to remove cellular RNA debris from dying cells could have acquired a selective anti-pathogen activity to provide the host protection against infection. An amoeboid-type secretory blood cell initially adapted to localized response and tissue repair duties would have acquired novel properties, allowing isolated tissue cells to communicate over an extended distance and participate in the overall systemic response (17).

## Role in Health and Disease

Overall, extracellular RNases display a variety of immune-related activities that ensure that the organism is fit for survival. The RNases participate in diverse tasks, from cellular housekeeping to ensuring the sterility of body fluids (Table 1). Following tissue damage by an external injury the RNases are expressed as alarm signaling molecules (54, 60, 61). Their secretion at the inflammation site contributes to tissue repair and remodeling (52, 62). To participate in the tissue healing process, the RNases can target and remove the host-damaged cells. Selective cytotoxicity can be mediated by the activation of autophagy or apoptotic pathways (72, 75). To facilitate subsequent tissue remodeling, the RNases also function as cytokines and chemokines, displaying anti-inflammatory activities and inducing chemoattraction of innate cells, such as macrophages or DCs (29, 46–48). Other complementary activities have been reported such as binding to nucleic acids, activation of TLR receptors and removal of exRNA (54, 55, 76).

RNases’ expression can also be induced during infections and the secreted RNases can directly participate in the killing of invading microorganisms (42, 84, 89) (Table 1). Overall, we observed an organized distribution of tasks among the distinct host innate cells that can ensure the coverage of wide spectra of potential pathogens. On the other hand, there is also a downregulation in RNases’ expression after extended periods of infection (Table 2). A close inspection of the RNases’ expression patterns suggests an adaptive process by the intracellular-dwelling pathogens to inhibit the host response and extend their survival lifespan.

Fortunately, extensive research on secretory RNases is currently setting the basis for applied therapies. Clinicians are already taking advantage of the selective secretion of RNases for monitoring and diagnosing inflammation. RNase3 levels are routinely used to monitor asthma processes (51). Levels of hRNase1, hRNase3, and hRNase7 are increased during sepsis and are proposed as markers for the diagnosis of organ failure (112). Another interesting proposal is the use of RI as a cancer biomarker (281).

Furthermore, successful results achieved using experimental animal models promise therapeutic applications in the near future. Removal of circulating exRNA shows beneficial anti-inflammatory properties following tissue damage (17, 29, 76). In particular, removal of blood exRNA can protect cardiac tissue in hypoxic conditions (29, 76). Treatment with hRNase1 has been observed to reduce deposits of exRNA and inflammation in a mouse model of atherosclerosis (78). RNases can also determine the fate of RNA stress granules. Under stress conditions, hRNase5 accumulates within stress granules (282). Local accumulation of RNA and alteration of RNA self-assembly is associated with neurodegenerative diseases (283). Selective cleavage of cellular RNAs mediates response to stress stimuli (56, 57). Overall, deregulation of non-coding RNA processing is a major cause of immune-malfunctioning and serious diseases (284).

Some RNaseA members can participate in biological functions such as hematopoiesis and angiogenesis, and show anti-tumoural properties mediated by selective cellular RNA targeting (6, 40, 285). The design of RNase constructs to develop specific immunotoxins that selectively target cancer cells is currently one of the most prioritize research topics. ImmunoRNases are engineered to be internalized by tumor cells, evade RI, and degrade cellular RNA (26, 269, 286–289). A recent nanocarrier delivery system using encapsulated RNaseA effectively achieves inhibition of cancer cell proliferation (290).

RNases can also maintain the sterility of biological fluids (100, 285). Eosinophils are involved in antiviral immunity and eosinophil RNases might mediate host response by TLR7 activation (55). Expression of hRNase2 is induced by HIV-1 infection and recombinant hRNase2 administration is proposed as an anti-HIV-1 therapy (100).

## Conclusion and Perspectives

The overview of the immuno-regulatory properties of secretory RNases highlights the similarities between their mechanisms of action and provides novel approaches to progress toward a deeper understanding of their ultimate *in vivo* biological role. We are confident that any step forward in this direction can consolidate our knowledge of the innate immune system and contribute to the development of novel treatments against immunological deregulations. In particular, the biological roles of RNases, such as in hematopoiesis regulation, tissue remodeling, prevention of infection and inflammation offer promising therapeutic applications.

## Author Contributions

LL, JL, MM, and EB contributed to the original draft and edited versions. LL and JL prepared the tables and graphical material. MM and EB wrote, edited, and revised the final manuscript version. All authors approved the final manuscript version.

## Conflict of Interest Statement

The authors declare that the research was conducted in the absence of any commercial or financial relationships that could be construed as a potential conflict of interest.
